# The Role of Oxidative Stress in TB Meningitis and Therapeutic Options

**DOI:** 10.3390/diseases12030050

**Published:** 2024-02-29

**Authors:** John Dawi, Aishvaryaa Shree Mohan, Yura Misakyan, Scarlet Affa, Edgar Gonzalez, Karim Hajjar, David Nikoghosyan, Sabrina Fardeheb, Christopher Tuohino, Vishwanath Venketaraman

**Affiliations:** 1 College of Osteopathic Medicine of the Pacific, Western University of Health Sciences, Pomona, CA 91766, USA; john.dawi@westernu.edu (J.D.); amohan@westernu.edu (A.S.M.); yura.misakyan@westernu.edu (Y.M.); edgar.gonzalez@westernu.edu (E.G.); karim.hajjar@westernu.edu (K.H.); david.nikoghosyan@westernu.edu (D.N.); sabrina.fardeheb@westernu.edu (S.F.); christopher.tuohino@westernu.edu (C.T.); 2 Los Angeles Valley College, Valley Glen, CA 91401, USA

**Keywords:** tuberculous meningitis, non-tuberculous meningitis, reactive oxygen species, ferroptosis, oxidative stress-induced TB meningitis

## Abstract

Meningitis is an inflammatory condition affecting the meninges surrounding the brain and spinal cord. Meningitis can be triggered by various factors, including infectious agents like viruses and bacteria and non-infectious contributors such as cancer or head injuries. The impact of meningitis on the central nervous system involves disruptions in the blood–brain barrier, cellular infiltrations, and structural alterations. The clinical features that differentiate between tuberculous meningitis (TBM) and non-tuberculous meningitis (NTM) are discussed in this review and aid in accurate diagnosis. The intricate interplay of reactive oxygen species, ferroptosis, and reactive nitrogen species within the central nervous system reveals a promising field of research for innovative therapeutic strategies tailored to TBM. This review highlights the alternative treatments targeting oxidative stress-induced TBM and ferroptosis, providing potential avenues for intervention in the pathogenesis of this complex condition.

## 1. Introduction: Meningitis Disease Process

Meningitis, characterized by inflammation in the meninges enveloping the brain and spinal cord, is occasionally referred to as spinal meningitis. The meninges are vital in shielding the brain and spinal cord, providing structural support and housing essential components like nerves, blood vessels, and cerebrospinal fluid. This inflammatory condition can be instigated by various factors, including infectious agents such as viruses and bacteria and non-infectious contributors like cancer or head injuries [1].

### 1.1. Molecular Bases of Meningitis Pathophysiology

Meningitis, irrespective of etiology, involves complex molecular interactions between the host and various infectious agents. The interplay of cytokines, chemokines, and immune cells orchestrates the inflammatory cascade. This section synthesizes the molecular underpinnings, emphasizing proteins, homeostatic structure, and compliance that govern the pathophysiological response. To begin with, the blood–brain barrier (BBB) relies on a complex interplay within the neurovascular unit, including brain endothelium, pericytes, astrocytes, and basal membranes. Brain endothelial cells maintain brain homeostasis through membrane transporters like glucose transporter 1 (GLUT1) and P-glycoprotein (Pgp), while tight junctions (TJs) prevent paracellular diffusion, ensuring cellular polarity. The glycocalyx on the luminal side and the basement membrane on the basolateral side contribute to barrier integrity. Brain microvascular endothelial cells exhibit a tight barrier, but post-capillary venules and veins have “leaky” junctions, potentially serving as a passage for pathogens into the cerebrospinal fluid [2,3,4,5].

Secondly, the blood–cerebrospinal fluid barrier (BCSFB) at the choroid plexus is formed by choroidal epithelial cells facing blood and cerebrospinal fluid (CSF). These cells control fenestration, allowing exchange between blood components and stromal tissue. ATP-binding cassette transporters (ABC), solute carrier transporters, and tight-junction complexes regulate barrier permeability. Choroidal epithelial cells produce CSF, and their strategic localization allows the integration of signals from blood and the brain. The choroid plexus contributes to neuroimmune surveillance and immune cell trafficking into the central nervous system [3,6].

**Figure 1:**
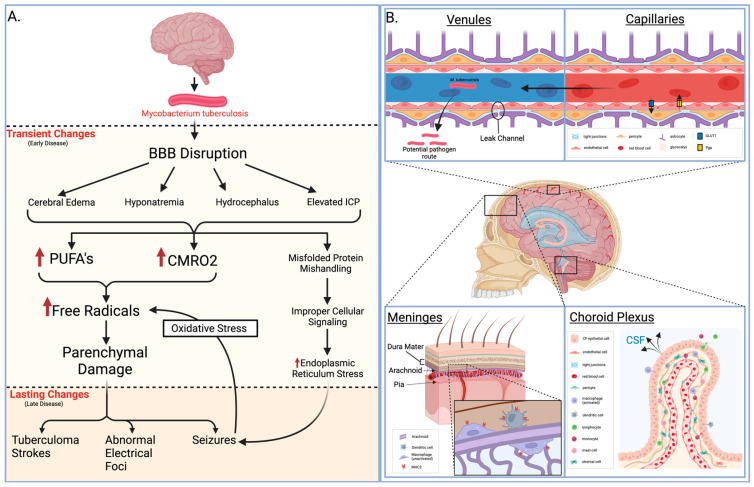
Pathology of meningitis. (**A**) Early and late pathological changes that result from *M. tuberculosis* meningitis. (**B**) Neurological and vascular structures displaying one infectious route, key immunological defenses, and relevant histology. BBB = blood–brain barrier, PUFA = polyunsaturated fatty acid, CMRO2 = cerebral metabolic rate of oxygen, GLUT1 = glucose transporter 1, Pgp = P-glycoprotein, MHC2 = major histocompatibility complex class II, CP = choroid plexus, CSF = cerebrospinal fluid.

In addition, the meninges covering the central nervous system (CNS) consist of the dura mater, arachnoid mater, and pia mater. The arachnoid mater primarily forms the BCSFB function, connecting leptomeningeal cells through tight junctions. Leptomeninges play a crucial role in bacterial meningitis, and an additional BCSFB function is proposed for pial vessels. Like the choroid plexus, the arachnoid regulates CNS immunity through major histocompatibility complex (MHC) class II-expressing myeloid cells. Macrophages and dendritic cells on the dura-facing side and resident macrophages on the CSF-facing side contribute to immune surveillance and potential antigen presentation to T cells [3,7,8,9] (Figure 1).

### 1.2. Pathology of Meningitis

Understanding the pathological changes induced by meningitis is crucial for unraveling its impact on the central nervous system. From disruptions in the BBB to cellular infiltrations, this section delves into histological aspects, shedding light on the structural alterations associated with meningitis. For example, pathogens like tuberculous meningitis (TBM) cause such a disruption, and seizures may occur due to various factors influenced by diverse pathological changes. Some of these changes are transient and can be resolved with appropriate measures, while others may persist, necessitating prolonged treatment with anti-epileptic drugs. Transient causes, often encountered in the early clinical phase, include meningeal irritation, cerebral edema, hyponatremia, hydrocephalus, and elevated intracranial pressure. In the later stages of TBM, multiple intracranial tuberculoma strokes, the development of abnormal electric foci, and other unidentified causes may contribute to seizures that pose challenges in terms of management. Seizures have the potential to induce oxidative stress in the brain parenchyma. The elevated cerebral metabolic rate of oxygen consumption (CMRO2) and the presence of polyunsaturated fatty acids (PUFAs) render the brain parenchyma vulnerable to lipid peroxidation and free radical injury (Figure 1).

In various central nervous system pathologies, excessive production of free radicals or diminished antioxidant activity can precipitate seizures, heightening the likelihood of recurrence. Conversely, seizures can also arise as a consequence of oxidative stress. Improper handling of misfolded proteins by the endoplasmic reticulum, leading to their accumulation, may result in cellular dysfunction and cell death by activating diverse signal transduction pathways. Impaired protein signaling can disrupt the translocation and transcription of proteins via pathways like the inositol pathway, which involves 1-α protein kinase-like ER kinase and activating transcriptional factor 6 (ATF-6). Endoplasmic reticulum stress (ER stress) is mitigated by unfolded protein response (UPR) through the involvement of the transcriptional factor X-box binding protein (XBP).

Seizures observed in TBM may be linked to heightened oxidative stress and endoplasmic reticulum (ER) stress, potentially influencing the severity of meningitis and impacting patient outcomes. Understanding the substantial impact of these changes in TBM could unveil potential therapeutic targets. Oxidative stress and ER stress concerning seizures associated with TBM have yet to be thoroughly examined [10].

### 1.3. US Statistics of Meningitis

In the United States, the annual toll of meningitis unfolds through more than 72,000 hospitalizations, incurring a substantial financial burden exceeding 1.2 billion USD [11]. Diverse strains of infectious meningitis cast shadows of heightened mortality and enduring complexities, encompassing neurological deficits and cognitive impairments [12,13]. Bacterial meningitis, a formidable adversary, wields mortality rates ranging from 10% to 20% in well-resourced settings and up to 50% in regions with fewer resources, where it assumes the somber rank of the fourth-leading cause of disability [12,13,14,15]. Conversely, aseptic meningitis, primarily of viral origin, generally presents a more optimistic prognosis, marked by a 4.5% mortality rate [16]. Tuberculous meningitis, on the other hand, unfurls a graver narrative, with mortality soaring to 50% in those grappling with HIV infection. Moreover, among survivors of tuberculosis meningitis, half navigate the aftermath burdened by neurological disabilities [13,17].

Parallel to this, fungal meningitis, notably cryptococcal meningitis, manifests with a poignant in-hospital mortality ranging between 30% and 50% [16]. On the global stage, Cryptococcus-induced meningitis bears the weight of 15% of AIDS-related deaths [18]. The specter of mortality also shrouds other rare forms of fungal meningitis. As Antinori et al. reported, Aspergillus meningitis carries a case fatality rate of 63.5% for those with intact immune defenses and a staggering 83% for immunocompromised patients [19]. Meningitis kindled by Coccidioides signals an unremitting journey toward mortality, hitting 90% at the first year’s end and an unrelenting 100% by the end of the second year without intervention [20]. Finally, central nervous system infections orchestrated by Histoplasma have a 39% case fatality rate [21].

### 1.4. Global Statistics of Meningitis

With the global goal of reducing meningitis by 2030, it has become a prevalent issue to defeat. According to the Global Burden of Diseases, Injuries, and Risk Factors Study (GBD), in 2019, there were an estimated 236,000 deaths due to meningitis, with the largest burden in children under the age of five, with 112,000 deaths [22]. Regions with the largest rate of meningitis include western sub-Saharan Africa, followed by eastern sub-Saharan Africa, central and south sub-Saharan Africa, and Oceania [23]. Globally, *S. pneumoniae* was the cause of the majority of meningitis cases, but in 2019, *N. meningitidis* had the highest concentration index [24]. Over the past 30 years, there has been an overall decrease in the number of deaths due to meningitis from 432,000 to 236,000 around the world, but the need for increased access to free immunization, prevention, and early diagnosis or treatment is still large [22,23]. From a database of 204 countries, those with lower life expectancy at birth, urban populations, and higher level of pollution were associated with a higher burden of meningitis [22].

Although globally, there is a downward trend in meningitis incidence, with 7.5 per 100,000 in 1990 to 3.3 per 100,000 in 2019, when looking at individual countries, the rates seem to be increasing, as in sub-Saharan Africa and Oceania [22]. As pediatric populations are the most susceptible, there is a higher prevalence among this age group and thus a higher mortality of 20–30% [23]. Further epidemiological evidence is needed to determine the exact pathogens that are currently responsible for the majority of meningitis cases worldwide. With this knowledge, further research can be undertaken to understand the pathophysiology and targets for effective therapeutics to move closer to the goal of defeating meningitis by 2030.

## 2. Tuberculous Meningitis and Non-Tuberculous Meningitis

Numerous pathogens can cause meningitis. This review attempts to differentiate between tuberculous meningitis (TBM) and non-tuberculous meningitis (NTM). Differentiating between the etiologies of meningitis can be challenging, but TBM has certain clinical features that can assist in diagnosis. These include positive CSF cultures for *Mycobacterium tuberculosis* (Mtb) and smears for acid-fast bacilli (AFB), basal enhancement on CT scan, and response to antituberculous treatment [25]. NTM primarily has bacterial, viral, or fungal etiology.

The disease process of bacterial meningitis involves several stages: mucosal colonization, systemic invasion, survival within the bloodstream and meninges, and neuronal damage due to increased intracranial pressure and altered cerebral blood flow [26]. The process begins with the colonization of the nasopharynx by the pathogen. This is followed by systemic invasion, leading to bacteremia. The bacterial encapsulation helps the pathogen resist phagocytosis and complement-mediated bactericidal activity, thus contributing to bacteremia. CNS invasion occurs after that, although the precise site of bacterial traversal into the CNS remains unknown. Bacterial replication and lysis in the subarachnoid space release virulence factors, inciting an inflammatory response in the CNS. This inflammation, marked by cytokine and chemokine release, leads to leukocyte influx, exacerbating brain damage. Studies have shown that pneumococcal cell wall components or Gram-negative bacteria’s lipo-oligosaccharides do not directly induce inflammation but do so via CNS-released mediators like interleukins and tumor necrosis factor [27]. These mediators increase BBB permeability, eventually leading to many pathophysiological consequences of bacterial meningitis, including cerebral edema and increased intracranial pressure.

In contrast, anti-inflammatory proteins like interleukin-10 (IL-10) and transforming growth factor beta 1 (TGF-β1) help regulate this inflammatory activity [28]. Clinically, bacterial meningitis patients often present with fever, headache, and signs of cerebral dysfunction. The classic triad of fever, neck stiffness, and altered mental status is not always present. The disease can progress to seizures, focal neurological deficits, and increased intracranial pressure, with pneumococcal and meningococcal meningitis occasionally presenting with rapid sepsis. Bacterial meningitis incidence varies significantly based on geographic and economic factors, ranging from about 0.9 cases per 100,000 individuals annually in high-income countries to 10–80 per 100,000 in low- and middle-income countries [29]. The mortality rates also differ widely, with adult and neonatal cases showing a range from 6% to 54%. Overall mortality varies from 10% in high-income regions, but can soar to 58% in low-income areas [30]. Bacterial meningitis constitutes roughly 13% of all adult meningitis and encephalitis cases in the United States. Among individuals aged over 16, Streptococcus pneumoniae accounts for 72% of bacterial meningitis cases, while Neisseria meningitidis accounts for 11% [31]. The most common causative agents in neonates are Streptococcus agalactiae (group B streptococcus), Escherichia coli, and Listeria monocytogenes [32].

Viruses responsible for meningitis are typically transmitted through inhalation, as seen with mumps, or ingestion, as with non-polio enteroviruses, and initially establish a primary infection in the body’s oropharyngeal or gastrointestinal lymphoid tissues. Once in the body, these pathogens can access the central nervous system (CNS) by several routes: they may infect cerebral vascular endothelial cells, cross the BBB by infecting hematopoietic cells, or travel via peripheral sensory or motor neurons. Upon reaching the CNS and establishing infection, the viral presence triggers the release of chemoattractants within the meninges, prompting an innate immune response marked by the infiltration of neutrophils, monocytes, and antiviral cluster of differentiation 8 (CD8) lymphocytes. This immune response to the viral invasion is a crucial feature of the pathogenesis of viral meningitis. Ultimately, the infection of the leptomeningeal cells leads to the clinical syndrome of viral meningitis. Patients with this condition typically present with fever, headache, and a stiff neck, considered hallmark symptoms of meningeal irritation [33]. The predominant cause of meningitis is viral in origin. Two extensive epidemiological studies by Dr. Rodrigo Hasbun and colleagues were carried out to explore the etiologies and outcomes of meningitis, examining adult cases and those in infants and children as distinct groups. In adult US populations diagnosed with meningitis or encephalitis, among the 26,429 patients identified, enteroviruses were the predominant etiology, responsible for 51.6% of all cases.

Fungal meningitis, although less common, is a significant concern, especially in immunocompromised individuals. It usually presents as a subacute or chronic process and can be as lethal as bacterial meningitis if left untreated. Most pathogenic fungi are inhaled, leading to a primary pulmonary infection, usually self-limited. Hematogenous dissemination may follow, with subsequent involvement of the CNS. The subarachnoid space and its contents are usually immunologically protected, with functional and anatomic barriers to invasion. In immunocompromised individuals, however, the dysfunctional protective structures allow penetration of fungal pathogens into the CNS [11]. Cryptococcal meningitis (CrM), predominantly caused by Cryptococcus neoformans or Cryptococcus gattii, is the leading cause of adult fungal meningitis and is increasingly a global health concern. HIV-infected individuals are at the greatest risk, with CrM implicated in up to 79% of cases within this group. CrM is responsible for a significant proportion of AIDS-related deaths—between 15% and 17%—even in regions with adequate health-care resources [34]. In a retrospective study by Charalambous et al., which reviewed 1927 US cases of fungal meningitis from 2000 to 2012, cryptococcal infections were identified as the cause in nearly 70% of instances. This group also experienced the highest health-care costs and mortality rates compared to other fungal etiologies of meningitis [11].

TBM’s global incidence and mortality are not well documented due to diagnostic difficulties and inconsistent reporting practices [35]. It is estimated that in 2019, approximately 164,000 adults worldwide developed TBM, with a significant 23% co-infected with HIV. Predominantly, cases were male (60%) and in the 25–34 age bracket (20%), with the majority of cases (70%) occurring in Southeast Asia and Africa. The study estimated that in the same year, 78,200 adults succumbed to TBM, which is about 48% of those afflicted [36]. The study, however, is not without limitations. Data variation due to different study designs, inclusion criteria, and TBM definitions could affect the estimates’ accuracy. The extrapolation of data to all countries may not account for local factors such as TB prevalence, population demographics, genetics, comorbidities, or the efficacy of health systems. The study also presumed that the proportion of undiagnosed TBM was equivalent to that of diagnosed cases, a supposition not backed by specific data, leading to potential inaccuracies. TBM stands as the deadliest manifestation of tuberculosis, and it is clear that there needs to be an improvement in TBM monitoring and treatment globally.

NTM and TBM present distinct pathological features and clinical progressions. NTM symptoms can vary, but include fever, headache, stiff neck, nausea, vomiting, photophobia, and altered mental status [37]. NTM, as we have seen, is most often caused by bacteria like Streptococcus pneumoniae and viruses such as enteroviruses, which lead to an acute inflammatory response in the meninges. The inflammation is typically characterized by rapid onset and, in bacterial forms, is associated with purulent CSF. At the same time, viral NTM generally results in a less severe lymphocytic pleocytosis with clear CSF. NTM can result in rare complications such as encephalitis and deep coma, but clinical recovery is generally rapid and effective treatment is established [38].

Conversely, TBM follows a more protracted course, often beginning with a subacute phase that may last for weeks. TBM symptoms include fever, headache, stiff neck, confusion, altered mental status, and neurological deficits [39]. The concept of a “Rich” focus, described by Rich and McCordock, seems to also play a part in TBM pathogenesis. They suggest that initial TB infection may lead to silent tuberculous lesions past the BBB. Afterward, activation of previously dormant tuberculous lesions releases the bacteria into the subarachnoid space, inciting a granulomatous infection of the meninges and consequential inflammatory response [40]. This inflammation can account for various clinical manifestations of TBM. For instance, inflammation around cerebral blood vessels, especially the middle cerebral artery, can lead to ischemia and cerebral infarcts. The spread of inflammation to the base of the brain may disrupt CSF circulation, causing hydrocephalus and increased intracranial pressure. Furthermore, the inflammatory exudates can envelop cranial nerves, leading to palsies, and the formation of expanding tubercles may result in tuberculomas or, more rarely, brain abscesses [41]. As such, TBM is more likely to cause severe neurological complications due to its chronic inflammatory process. Another hallmark of TBM pathology is thick, gelatinous exudates in the basal subarachnoid space, leading to obstructions in CSF flow and hydrocephalus. Finally, TBM may be characterized by the formation of granulomas and infarcts due to endarteritis [41]. As TBM and NTM can present with similar symptoms due to the inflammation of the brain and spinal cord, it is important to understand the specific pathology and pathways that are affected to provide targeted and effective treatment.

## 3. Oxidative Stress in Tuberculosis

TB and NTM pose significant global health challenges due to their intricate pathogenesis and the complex interplay between infecting agents and the host’s immune response. Recent scientific endeavors have shed light on the crucial role of oxidative stress and reactive species in these infectious diseases. Oxidative stress, mediated by reactive oxygen species (ROS), ferroptosis, and reactive nitrogen species (RNS), has emerged as a key contributor to the progression, severity, and modulation of the immune response in TB and NTM.

Reactive oxygen species (ROS) play a critical role in bolstering the host’s defense against TBM. However, the TB-causing Mycobacterium tuberculosis (Mtb) has developed mechanisms to counteract and evade the effects of ROS. Particularly within the CNS, Mtb produces antioxidant enzymes like superoxide dismutase, catalase-peroxidase, and alkyl hydroperoxide reductase. These enzymes effectively neutralize ROS, including superoxide radicals and hydrogen peroxide, prevalent within the CNS environment. By breaking down ROS, the bacteria can thrive amidst the oxidative stress within the CNS [42]. Moreover, Mtb possesses NADH dehydrogenase enzymes that assist in evading ROS production by enabling electron transport without substantial superoxide generation. Additionally, the mycolic acid in the cell wall of Mtb acts as a protective shield against ROS, serving as a barrier amid oxidative stress conditions within the CNS [43].

Ferroptosis, a newly explored phenomenon, is gaining attention in the realm of tuberculous meningitis. Ferroptosis, an iron-dependent form of regulated cell death characterized by lipid peroxidation and the accumulation of toxic lipid reactive intermediates, is being increasingly studied within the context of TBM. Mtb has evolved mechanisms to acquire iron from the host within the CNS, leading to an accumulation of excess iron, thereby creating a conducive environment for ferroptosis [44]. Among the seven identified glutathione peroxidase (GPX) enzymes, GPX4 is most closely associated with the ferroptosis pathways [45]. Studies have emphasized the significance of GPX4 with ferroptosis, as depletion of GPX4 has shown lipid peroxidation-induced cell death in mouse brain cells [45]. Studies have also shown the direct relationship between the inactivation of GPX4 leading to a toxic increase of ROS, leading to ferroptosis [46]. Investigating the major role of GPX4 in ferroptosis pathways can provide insight into novel therapeutics for various pathologies. A recent study demonstrated that Mtb infection decreased GPX4 and increased lipid peroxidation in host cells. Mtb infection caused necrotic cell death in culture, and the pulmonary pathology in vivo was alleviated by ferroptosis inhibitor treatment.

Moreover, the study revealed that Mtb lacking protein tyrosine phosphatase A (PtpA), which serves as a pro-ferroptosis factor, exhibited a reduced capability to lower GPX4 expression compared to the wild-type Mtb. This finding further supports that PtpA is a crucial effector in Mtb, inhibiting GPX4 expression and encouraging ferroptosis in host cells [47,48]. However, the detailed interplay between ferroptosis and TBM remains a subject of ongoing research, requiring further exploration for a comprehensive understanding.

**Figure 2:**
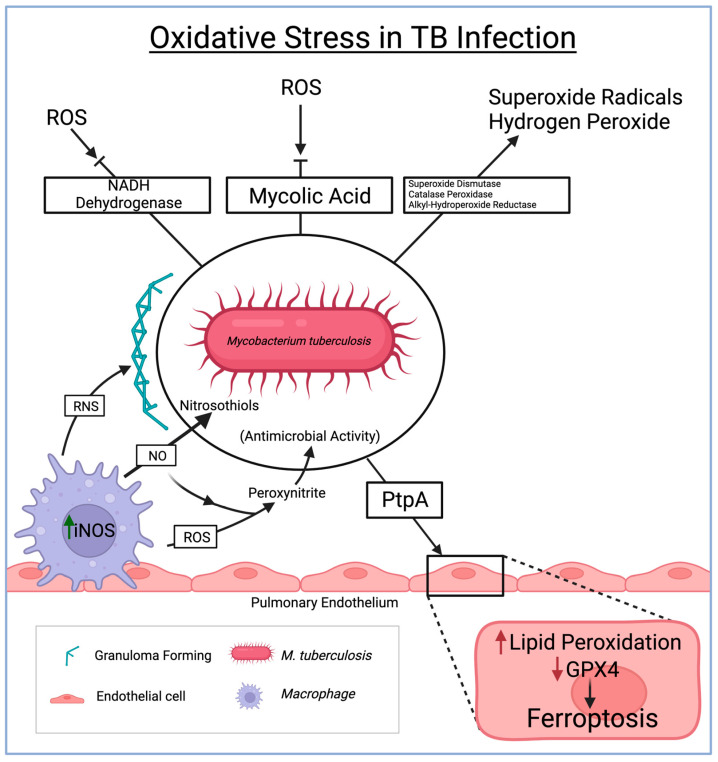
The role of oxidative stress in the immune system and consequent defenses by *M. tuberculosis* in driving pathological routes to endothelial damage and granuloma formation. ROS = reactive oxygen species, TB = tuberculosis, NADH = nicotinamide adenine dinucleotide + hydrogen, RNS = reactive nitrogen species, PtpA = protein tyrosine phosphatase A, GPX4 = glutathione peroxidase 4.

Reactive nitrogen species (RNS) also significantly contribute to the immune response against Mtb within the CNS during TBM. Nitric oxide, a key RNS, reacts with superoxide anions to form highly reactive nitrogen intermediates such as peroxynitrite [39]. These intermediates function as potent antimicrobial agents, inflicting damage upon Mtb. Furthermore, nitric oxide disrupts Mtb survival mechanisms by reacting with thiol groups in proteins, leading to the formation of nitrosothiols. RNS also plays a pivotal role in granuloma formation (the organized aggregation of immune cells containing Mtb within the CNS), which is crucial in preventing the spread of TBM [49] (Figure 2).

The relationship between tuberculous meningitis and the intricate interplay of reactive oxygen species, ferroptosis, and reactive nitrogen species within the central nervous system represents a burgeoning field of research. Understanding these complex interactions is paramount for developing innovative therapeutic strategies tailored to TBM. Delving deeper into these mechanisms holds promise for elucidating novel avenues to intervene in the pathogenesis of tuberculous meningitis.

## 4. TB Meningitis Treatment Options

In order to effectively treat TBM, it is imperative to examine three components for successful management: (i) rapidly diminishing the actively multiplying bacilli to reduce disease severity, mortality, and transmission, (ii) eliminating populations of lingering bacilli to ensure a lasting cure and prevent relapse, and (iii) averting the development of drug resistance throughout treatment [50]. Initiating treatment for TBM is crucial at the earliest signs of clinical suspicion, even before obtaining microbiological confirmation through molecular tests, mycobacterial culture, and acid-fast bacilli smear microscopy [50,51]. According to the WHO, a patient should initially take at least five drugs, including fluoroquinolone and an alternative injectable agent, for 18–24 months [52]. The traditional chemotherapy regimen, also known as the RIPE protocol, involves an “intensive phase” with four drugs, followed by an extensive “continuation phase” with two drugs [53]. Precisely, it consists of two months of isoniazid (INH) 5 mg/kg/day (~300 mg/day), rifampicin (RMP) 10 mg/kg/day (~450 mg/day), pyrazinamide (PZE) 25 mg/kg/day (~1500 mg/day), and streptomycin (SM)/ethambutol (ETB) 15 mg/kg/day (~800 mg/day) followed by INH and RMP for 7–10 months [50,51,52,54].

In all forms of tuberculosis, INH serves as a crucial chemotherapeutic agent in the treatment of TBM, as it quickly reaches the CNS, rapidly reduces mortality, and demonstrates robust bactericidal activity [51,52,53]. In contrast, RMP does not freely penetrate the BBB as effectively, with 10–20% of its concentration in the CSF, and only becomes activated when unbound to protein [52]. However, patients with RMP-resistant strains experience an increased mortality to TBM, confirming its role in the treatment protocol [51]. PZE has effective CNS penetration, with similar concentrations in the CSF and serum, and reduces the duration of treatment for drug-susceptible TB [51,52]. Although its bactericidal activity is diminished in the first 2–4 days of treatment, it reaches potency similar to INH and RMP in days 4–14 [52]. The last of the RIPE regimens, SM or ETB, has the poorest CNS penetration and provokes significant adverse effects with long-term use, which leads researchers to investigate the use of a fourth drug [51,52,53]. (Table 1) However, with the significant rise in RPM-resistant (RR) and multidrug-resistant TB (MDR-TB), further research is implicated to successfully optimize patient outcomes.

Although antibiotic therapy overall reduces morbidity, its variable penetration in the brain and severe inflammatory response renders it ineffective at curing TBM [55]. In addition to the RIPE protocol, fluoroquinolones are incorporated to control TBM disruption and mitigate multidrug-resistant cases. The newer generation of fluoroquinolones, such as levofloxacin and moxifloxacin, exhibited highly potent activity against most Mtb strains, excellent CSF penetration, and favorable safety profiles [51]. In a randomized study, 61 patients were assigned to one of two groups for the first month of treatment: a control group of standard treatment alone or standard treatment with ciprofloxacin (750 mg every 12 h), levofloxacin (500 mg every 12 h), or gatifloxacin (400 mg every 12 h) [52,56]. When the medications were used prior to coma onset, results demonstrated increased survival rates, reduced disability burden, and lower incidence of disease relapse [52]. In a follow-up study conducted in Indonesia, researchers administered either an elevated (600 mg) or regular (450 mg) dosage of rifampicin and either an elevated (800 mg) or regular (400 mg) dosage of moxifloxacin among 60 adults diagnosed with TBM [57] (Table 1). In addition to the benefits in treatment through the administration of an elevated dosage of moxifloxacin, high-dose rifampicin led to elevated levels in both plasma and CSF and was linked to lowered mortality rates (65% compared to 35%) [57]. The higher-dosage treatment regimens resulted in a proportional increase in CSF penetration with no increase in toxicity, demonstrating the possibility of combination treatment with fluoroquinolones in cases of multidrug resistance [57]. Although fluoroquinolones serve as an effective addition to the standard anti-TB regimen, an optimal dose regimen is yet to be established.

The neurological aspects of TBM manifest as an overactive inflammatory response that extensively damages tissue and confines the brain to a fixed space [51,55]. Therefore, there has been considerable attention on supplementary host-directed immune interventions to boost protective immunity, limit neurological complications, or modulate tissue destruction [52,58]. Among these interventions, corticosteroids are the most extensively studied host-directed therapy to prevent the generation of proinflammatory markers [51,52,55]. Some benefits include limited tissue necrosis, granuloma destruction, enhanced drug penetration, amplified bacterial clearance, and decreased likelihood of relapse [59].

Recent research has specifically evaluated the genetic polymorphism in the leukotriene A4 hydrolase (LTA4H) promoter, which regulates the equilibrium between proinflammatory and anti-inflammatory eicosanoids, consequently impacting the expression of tumor necrosis factor (TNF) alpha [52,56]. Understanding the role of LTA4H can provide insight into how effective or harmful the addition of corticosteroids can be for a patient with TBM. In a retrospective analysis, scientists enrolled TBM patients in a trial of anti-tuberculosis therapy and supplementary dexamethasone [52]. Patients with the TT genotype (hyperinflammatory) for LTA4H had enhanced survivability with dexamethasone, whereas patients with the CC genotype (hypoinflammatory) had non-beneficial or harmful effects [52]. This study suggests that dexamethasone plays a critical role as an adjunctive therapy in suppressing inflammation and supporting individualized immunotherapy with pretreatment genotyping for TBM [52,58]. Further investigation in pharmacogenetics regarding TBM can provide a more personalized and precise approach to achieving effective treatment and increased survival.

In a comprehensive study conducted in Vietnam, various doses of dexamethasone were tapered over 6–8 weeks depending on age and disease severity. For children, the treatment regimen involved dexamethasone at a dosage of intramuscular (IM) 12 mg/day for three weeks, followed by a gradual tapering over the subsequent three weeks [51]. Individuals with mild disease were provided with intravenous (IV) dexamethasone at a rate of 0.3 mg/kg/day for one week, followed by 0.2 mg/kg/day for another week, and then four weeks of gradually decreasing oral therapy [51]. For patients experiencing more severe TBM, IV dexamethasone was administered for four weeks, with a weekly dosage sequence of 0.4 mg/kg/day, 0.3 mg/kg/day, 0.2 mg/kg/day, and 0.1 mg/kg/day, followed by an additional four weeks of tapering oral dexamethasone therapy [51]. Nine months after treatment, patients followed up showed reduced risk of death and frequency of adverse effects, such as hepatitis, which proves its efficacy alongside the anti-TB drug regimen [51,53,58]. Corticosteroids also decreased basal meningeal inflammation, brain-stem encephalopathy, and the incidence of drug hypersensitivity reactions [58]. Although adjunctive corticosteroid treatment is accompanied by immunosuppression and does not completely avert disease morbidity, it manages exacerbated inflammation and mitigates the pathogenesis of TBM [55,58]. Nevertheless, additional research is necessary to explore the safety and effectiveness of rigorous treatment protocols and novel anti-tuberculosis agents for addressing tuberculosis meningitis.

## 5. Alternative Therapy for Oxidative Stress-Induced TB Meningitis

The management of TBM relies on eliminating Mtb and managing host inflammatory responses [60]. Alternative treatment of oxidative stress-induced TB meningitis heavily depends on eliminating inflammatory processes such as ferroptosis. As previously established, oxidative stress induces ferroptosis through the overload of reactive oxygen species (ROS), which leads to lipid peroxidation and glutathione (GSH) depletion; hence, ferroptosis plays a key role in oxidative stress-induced TB meningitis [61]. As the disease takes its course, ferroptosis simultaneously occurs. For this reason, researchers have explored various alternative therapies for the treatment of oxidative stress-induced TB meningitis, which directly target the ferroptosis and oxidative stress mechanisms underlying the illness.

Iron chelators are a prevalent and continuously studied therapy for ferroptosis. The FDA has approved three main iron chelators for clinical administration: deferoxamine (DFO), deferiprone (DFP), and deferasirox (DFX) [62]. Iron chelators such as DFO or DFP work by binding to the accumulated iron to prevent mitochondrial ROS accumulation [63]. They can also remove iron from lipid-peroxidizing enzymes, specifically lipoxygenases (LOXs), which are fundamental to ferroptosis mechanisms [63]. As a result, they prevent further ferroptosis by eliminating and preventing iron overload [63]. Since DFO has low oral absorbability, it is administered intravenously or intramuscularly [63]. It is administered through a subcutaneous infusion 5–7 nights a week or through an intravenous line for 24 h [63]. The dosage for adults is within 1–2 g/kg and 20–40 mg/kg for pediatric patients [63]. This treatment is specifically for iron overload and ferroptosis by extension. A recent study corroborated the continuous use of DFO as the best treatment for transfusion iron overload [63].

Nonetheless, DFO poses numerous clinical limitations and side effects, such as skin, ocular, and auditory reactions, with neurological and pulmonary disorders being observed at high doses [63]. DFP, on the other hand, is orally administered in tablet or solution form. The dosage for adults and pediatric patients is 75 mg/kg per day divided over three separate doses. The recommended dose is 100 mg/kg daily [63]. Studies indicate that integrated treatment of DFP and DFO or DFP and DFX should be carefully and cautiously examined before application. Despite posing health risks such as gastrointestinal symptoms and agranulocytosis, DFP is better than DFO since it penetrates the lipid membrane more efficiently, eliminating iron overload and decreasing the risk of continued oxidative stress [62,63]. Finally, DFX, orally administered, has shown better results than DFO and DFP [64]. The highest recommended dosage is 30 mg/kg per day, and it is given in one of two forms: DFX film-coated tablets (FCT), a newly approved form, or DFX dispersible tablets (DT). Though it is convenient due to its once-daily formulation, DFX is relatively expensive and can be unattainable for a number of patients [63]. These three iron chelators have proven to reduce and eventually eradicate iron overload and ferroptosis. Treating ferroptosis through iron chelation therapy has also been proven to reduce Mtb viability, further supporting the use of iron chelators for oxidative stress-induced TB meningitis [65].

A recent study supports the use of deferric amine compounds (DFAs), new iron chelators, for treating iron overload and preventing ferroptosis [64]. The study analyzed seven different DFAs and observed that DFA1 demonstrated greater efficiency in iron chelation than the three previously discussed iron chelators [64]. DFA1 was administered intravenously in 30 mg/kg doses every other day for two weeks [64]. Orally, it was administered in 20 mg/kg doses once every other day for four weeks [64]. Reducing the protein levels of major regulators of ferroptosis, such as L-ferritin light (FTL) and NADPH oxidase 1 (NOX1), and increasing the concentration of GPX4, DFA1 proved its efficacy as an anti-ferroptosis iron chelator [64]. It is important to note that since these iron chelators are relatively new, the study was conducted on mice. Therefore, extensive research must be conducted on the use of DFAs in human patients with iron overload and oxidative stress-induced TB meningitis.

Furthermore, GSH depletion plays a crucial role in ferroptosis and oxidative stress-induced TB meningitis, so GSH therapy is currently being researched as an alternative therapy for oxidative stress and ferroptosis by extension. GSH, the cell’s primary antioxidant, has been proven to combat the formation of destructive free radicals, reducing oxidative stress [66]. GSH acts explicitly as a hydroxyl radical scavenger [67]. A recent study conducted on the role of GSH in the reduction of oxidative stress found that cells with cytotoxicity induced by treatment of 500 μM H2O2, which were treated with 0.8 mM, 1.6 mM, and 3.2 mM glutathione for 1 h prior to the H2O2 treatment, showed significantly greater cell viability after 24 h [68]. As the concentration of GSH increased, so did the viability of the cells [68].

Along with inhibiting cytotoxicity, GSH helps suppress cell death [68]. That study maintained that GSH activates the nuclear factor erythroid 2-related factor 2 (Nrf2)/heme oxygenase 1 (HO-1) signaling pathway, essential in combating oxidative stress and cell death [68]. Additionally, the supplementation and enhancement of the GSH system through direct administration in vivo has been recently reported to increase GSH levels and hinder oxidative stress mechanisms [69]. Another study found that supplementing precursors of GSH, such as glycine, glutamate, cysteine, and selenium, enhances GSH levels and reduces oxidative stress markers [70]. Given the extensive research on the link between GSH and oxidative stress, it is clear that GSH positively affects the reduction of oxidative stress and ferroptosis by extension. However, GSH therapy has yet to be as extensively studied as iron chelation therapy in terms of its link to oxidative stress-induced TB meningitis. Therefore, more research needs to be conducted specifically on the dosing of GSH supplements and treatment protocols for patients with oxidative stress-induced TB meningitis.

Saffron, a Mediterranean spice plant known as Crocus sativus, has also proven effective in combating oxidative stress and its harmful effects [71]. Saffron is extremely rich in antioxidants, and this capacity contributes to its efficacy regarding oxidative stress inhibition [71]. A meta-analysis of 10 controlled trials further clarified saffron’s role in oxidative stress prevention [71]. One of the studies analyzed concluded that saffron increased the radical scavenging activity of diphenyl picryl hydrazyl (DPPH) by acting as an antioxidant and donating a hydrogen atom to the DPPH radical anion [71]. This supports the overall findings that biological markers of oxidative stress, such as malondialdehyde (MDA) levels and total antioxidant capacity (TAC), were impacted in a way that reduced oxidative stress [71]. However, according to another study within the meta-analysis, F2-isoprostanes, which are the critical substances of lipid peroxidation and ferroptosis, were not impacted by saffron intake [71]. Therefore, some oxidative stress markers require additional random controlled clinical trials (RCTs) to understand saffron’s effects fully. The meta-analysis revealed that patients who received doses of 50 mg/kg per day or greater of saffron had lower MDA levels and higher TAC levels [71]. This was especially true for patients with intervention plans lasting 10 weeks or less [71]. Interestingly, saffron intake was more effective for middle-aged than senior participants [71].

Moreover, another meta-analysis of 16 RCTs concluded that patients who received doses less than 30 mg/kg daily and for a period shorter than 12 weeks demonstrated significant decreases in MDA levels [72]. Other markers of oxidative stress, such as total oxidant status (TOS), TAC, glutathione peroxidase (GPX), superoxide dismutase (SOD), and prooxidant–antioxidant balance (PAB), were also impacted in an oxidative stress-reducing manner. However, the conditions under which this occurred contradicted previously outlined treatment protocols [72]. Given this ambiguity, RCTs with larger samples must be carried out to assess saffron’s effects on various oxidative stress markers, its optimal dosing, and its role in therapeutic endeavors for oxidative stress-induced TB meningitis.

## 6. Conclusions

In conclusion, this comprehensive review has elucidated the distinct features and clinical progressions of tuberculous meningitis (TBM) and non-tuberculous meningitis (NTM), shedding light on the challenges in their diagnosis and treatment. Bacterial meningitis, predominantly caused by Streptococcus pneumoniae and Neisseria meningitidis, carries varying mortality rates and incidence worldwide. Viral meningitis, particularly enterovirus-induced, prevails in adult populations. Fungal meningitis, notably Cryptococcal meningitis, poses a significant threat, especially in immunocompromised individuals. The paper highlights the global impact of TBM, emphasizing its diagnostic difficulties and the urgent need for improved monitoring and treatment strategies. The role of oxidative stress in tuberculosis, specifically the interplay of reactive oxygen species (ROS), ferroptosis, and reactive nitrogen species (RNS), has been explored, providing insights into potential therapeutic interventions. Treatment options for TBM involve a multidrug approach including fluoroquinolones and corticosteroids, with a focus on early initiation. The complexity of TBM necessitates a continuous exploration of alternative therapies targeting oxidative stress-induced pathways. Iron chelators, glutathione therapy, and saffron supplementation emerge as potential avenues for further investigation. Future research should delve into refining diagnostic methods for TBM, exploring innovative treatment combinations, and evaluating the safety and efficacy of alternative therapies. Developing a nuanced understanding of the host–pathogen interactions and the impact of oxidative stress in TBM will be crucial for advancing therapeutic strategies and improving patient outcomes.

## Figures and Tables

**Table 1:** Summary of treatment options for TB meningitis and key features.

Treatment	Dosage (Experimental)	Effect/Key Features
Antibiotics		
Isoniazid (INH)	5 mg/kg/day (~300 mg/day)	High CSF penetration
Rifampicin (RMP)	10 mg/kg/day (~450 mg/day)	High/Moderate CSF penetration
Pyrazinamide (PZE)	25 mg/kg/day (~1500 mg/day)	Moderate CSF penetration
Streptomycin (SM)	15 mg/kg/day (~800 mg/day)	Low CSF penetration
Ethambutol (ETB)	15 mg/kg/day (~800 mg/day)	Low CSF penetration
Fluoroquinolones		
Levofloxacin	500 mg every 12 h	High potent activity High CSF penetration
Ciprofloxacin	750 mg every 12 h	Increased survival rates Reduced disability burden Lower incidence of disease relapse
Gatifloxacin	400 mg every 12 h	Increased survival rates Reduced disability burden Lower incidence of disease relapse
Moxifloxacin	400 mg every 12 h	Highly potent activity High CSF penetration
Corticosteroids		
Dexamethasone	Varies based on disease severity	Reduced risk of adverse effects Suppresses inflammation Decreased brain-stem encephalopathy

## Data Availability

Data are available within the references.
